# Bipolar Disorder in Social Media: An Examination of Instagram's Role in Disseminating Accurate Information

**DOI:** 10.7759/cureus.46296

**Published:** 2023-09-30

**Authors:** Prachi Patel, Manasi Nagare, Jaismeen Randhawa, Abid Ali, Laura Olivieri

**Affiliations:** 1 Medicine and Surgery, Rajarshee Chhatrapati Shahu Maharaj (RCSM) Government Medical College, Kolhapur, IND; 2 Internal Medicine, Smt Mathurabai Bhausaheb Thorat (SMBT) Institute of Medical Sciences and Research Centre, Nashik, IND; 3 Psychiatry, Sri Guru Ram Das Institute of Medical Sciences and Research, Amritsar, IND; 4 Internal Medicine, Khyber Medical College, Peshawar, PAK; 5 Internal Medicine, University of New England College of Osteopathic Medicine, Biddeford, USA

**Keywords:** social media, reliability score, global quality score, instagram, bipolar disorder

## Abstract

Introduction

Bipolar disorder is a chronic and recurring condition marked by fluctuations in both energy and mood that can be debilitating to individuals without treatment. While physicians clinically diagnose the condition, patients frequently seek information from alternate channels. Social media platforms, such as Instagram, have facilitated more convenient access to supplementary information about bipolar disorder. Nevertheless, there is apprehension regarding the accuracy of publicly disseminated information through these platforms. The aim of this study is to evaluate the accuracy and dependability of information about Bipolar disorder found on the social media platform, Instagram.

Methodology

A cross-sectional observational study was conducted by gathering data from Instagram posts linked with popular bipolar disorder hashtags. To evaluate the credibility of the chosen entries, numerical ratings were assigned to each post using the established measurement scales of the Global Quality Score and Reliability Score.

Results

After analyzing 196 Instagram entries about Bipolar Disorder that fulfilled inclusion criteria, the study revealed that 70.4% (n=138) of these posts were shared by individuals diagnosed with bipolar disorder. Among the content posted by these individuals, a statistically significant global quality score of 2 and a reliability score of 1 were observed.

Conclusions

Verified medical information of superior global quality should be shared on social media platforms by accountable parties. Individuals with further inquiries about medical knowledge should be advised to reach out to local physicians.

## Introduction

Bipolar disorder is a chronic recurring illness characterized by swings in energy and mood. It affects more than 1% of the world's population. One of the main causes of disability in young people is bipolar illness, which can result in cognitive and functional impairment as well as increased mortality, notably suicide. Because the development of bipolar disorder typically coincides with a depressive episode and resembles unipolar depression, accurate diagnosis of the condition is challenging in clinical settings. Furthermore, the condition currently lacks any reliable biomarkers. Therefore, clinical assessment continues to play a crucial role [1]. The use of atypical antipsychotics, lithium, and valproate has been commonly indicated in the management of manic episodes; furthermore, lamotrigine, olanzapine, and lithium have also been proven to be significant as alternatives for maintenance therapy, as per the guidelines stated by Fountoulakis et al. [2].

Social media is a wealth of information and an easily accessible platform for wide varied information. It is an elaborate branch where individuals prefer to search for the disease, its symptoms, treatments and prognosis far before even approaching the doctor [3]. Because of the ease of use at the comfort of the home, it is utilized to establish connections and provide priority services.

The relationship between bipolar disorder and social media is complex and has both positive and negative aspects. Social media platforms provide a community through groups, pages can help connect with others going through comparable difficulties, discuss coping mechanisms, and offer emotional support. A better understanding of the condition enables the patient to make informed decision and overcome the stigma of mental health. The truth that people share on social media is sometimes misrepresented since they only share their highlights, thereby exacerbating depressive episodes. Social media usage for validation and affirmation lowers self-esteem and causes an emotional need for virtual approval.

The aim of this study is to assess the quality and reliability of information related to bipolar disorder on the social media platform, Instagram, using the Global Quality Score (GQS), the reliability score adapted from DISCERN.

## Materials and methods

This study is a cross-sectional observational study conducted in August 2023. Data was gathered from Instagram over two days, specifically on August 9th and 10th.

Each author was assigned one of the following hashtags, #bipolar, #bipolarawareness, #bipolardisorder, #bipolarrecovery, and #bipolarwarrior, and the top 100 posts for each hashtag were analyzed. A premade questionnaire was used by all authors to collect data. Posts in the English language that were relevant to bipolar disorder were included in the analysis. Posts in language other than English and not relevant to bipolar disorder were excluded from the study. Various aspects of the posts, including their distinct characteristics and the content, were thoroughly analyzed. These characteristics encompassed the type of post, such as whether it was an image or a video/reel, alongside metrics quantifying audience engagement, including the number of likes, comments, and followers. Additionally, the study examined the uploader's profile, distinguishing between doctors, hospitals, healthcare organizations, survivors/patients, or other entities.

Furthermore, the content of the posts was thoroughly evaluated, including different types of information shared, such as descriptions of symptoms, prevalence/incidence data, etiological details, diagnostic information, vaccine/shot-related content, treatment insights, mortality data, rehabilitation information, and details about support groups. Additionally, each post was classified based on whether it featured a digitally crafted image/video and whether any promotional content from pharmaceutical companies or doctors was present.

The assessment of the selected posts was then performed utilizing the GQS (Global Quality Score) and reliability was checked using the DISCERN score [3]. The collected data was transferred to a Microsoft Excel sheet for statistical analysis using SPSS version 21 (IBM Corp., Armonk, NY, USA). The Kruskal-Wallis test was employed to determine the association between the GQS and DISCERN scores.

## Results

For this study, 500 posts were analyzed overall. After applying inclusion and exclusion criteria, a total of 196 posts were considered for further analysis.

Table 1 describes the total number of posts included with their respective hashtags. This table shows that many posts with these hashtags don’t fit the inclusion criteria and thus get excluded. The highest number of inclusion posts were of #bipolarwarrior (n=77).

**Table 1:** Total posts evaluated and included in the study.

S.no	Hashtag	Posts analysed	Posts Included
1	#bipolar	100	12
2	#bipolarawareness	100	46
3	#bipolardisorder	100	16
4	#bipolarrecovery	100	45
5	#bipolarwarrior	100	77
	Total	500	196

Table 2 gives comprehensive information about the uploader, the type of post, and the size of the audience reached. Most of the posts were images, and the total number of likes was 349,409 with 3,379 comments, with the total number of followers of each account totaling to 5,125,083. The most common type of uploaders were patients (70.4%) and the least common uploaders were medical professionals (4.6%).

**Table 2:** Characteristics of posts. The comprehensive information about the uploader, type of post and the size of audience reached.

Type of posts	N (%)
Image	160 (81.6%)
Video / Reel	36 (18.4%)
Total number of audience reached	
Number of likes	349,409
Number of comments	3379
Number of followers	5,125,083
Type of uploader	
Medical Professional	09 (4.6%)
Healthcare Organization	23 (11.7%)
Patient	138 (70.4%)
Others	26 (13.3%)

Table 3 gives a thorough overview of the type of information shared via the posts. The three most common patterns seen in the posts were digitally created images or videos (62.24%), descriptions of symptoms (61.73%), and people/patients sharing their own experiences (55.61%). Very few posts had promotional content by pharmaceutical companies or by doctors (4.08%).

**Table 3:** A thorough overview of the type of information shared via the posts.

Criteria	N (%)
Description about the disease (Explaining what is it)	67 (34.18%)
Description of symptoms	121 (61.73%)
Information about prevalence/incidence	22 (11.22%)
Information about cause/etiology	10 (5.1%)
Information about diagnosis	28 (14.29%)
Information about prevalence	21 (10.71%)
Information about treatment	37 (18.88%)
Information about mortality	11 (5.61%)
Information about rehabilitation	28 (14.29%)
Information about support groups	12 (6.12%)
Information about people/patients sharing their own experiences	109 (55.61%)
Information about parent sharing their experiences with family members	17 (8.67%)
Is it a digitally created image/video?	122 (62.24%)
The post has promotional content by pharmaceutical companies or by doctors	8 (4.08%)

Table 4 assigns a Global Quality Score (GQS) and Reliability score (DISCERN) to the type of uploader to assess the quality and reliability of the posts. The respective median Global Quality Score (GQS) for posts by uploader was highest for medical professionals, with a score of 3 (2,4), followed by healthcare organizations, with a score of 2 (2,3), patients having a score of 2 (1,2) and others with a score of 2 (1,3.25). The p-value was found to be 0.007, and thus, the difference is statistically significant (P-value < 0.05).

**Table 4:** Assessment of quality and reliability of posts based on uploader. Values are written as median (Q1, Q3) where Q is Quartile. P-value < 0.05 is significant.

Type of Uploader	Global Quality Score	Reliability score (DISCERN)
Medical Professionals	3 (2, 4)	1 (1, 2)
Healthcare Organization	2 (2, 3)	2 (1, 3)
Patient	2 (1, 2)	1 (1, 2)
Others	2 (1, 3.25)	1 (1, 2)
P-value (Method: Kruskal-Wallis Test)	0.007	0.053

Median Reliability score (DISCERN) for posts by uploader was highest for healthcare organizations with a score of 2 (1,3) followed by medical organizations, patients and others, each having a score of 1 (1,2). Here, the p-value was found to be 0.053, and hence the difference is not statistically significant.

## Discussion

In our study, a total of 196 Instagram posts that met inclusion criteria were analyzed. These posts had a combined 349409 likes and 3379 comments. Around 138 (70.4%) of the posts were uploaded by patients suffering from bipolar disorder and the rest by medical professionals and healthcare organizations. Around 121 (61.73%) of the posts described the symptoms of bipolar disorders and 37 (18.88%) about treatment. The quality of posts uploaded by medical professionals was of higher quality compared to posts uploaded by patients, as assessed with GQS and this difference was significant.

In all social media platforms like YouTube, Instagram, or Twitter, information is created, condensed, and subsequently conveyed as content through many discourses and narratives that can reach a large number of audiences around the world. It is recognized as a medium that people can use to improve their health [4]. Users, for instance, can encourage others to follow their lead, resulting in increased levels of well-being, or they can help people connect with others who share their interests. Studies have also shown that postings on social media lessen the stigma attached to mental health problems [5,6]. While the accompanying content may include true elements and details that define the topic, it may also include modified, incorrect, or irrelevant information that alters how users perceive information and the behaviors and beliefs that go along with it [7, 8]. Thus, it is important to understand the accuracy and dependability of the information available on these platforms. In this article, we analyzed and presented the reliability and quality of user-generated content on Bipolar Disorder (BD) on Instagram, which is one of the most popular social media platforms.

It has been observed that some people share images, videos, or reels on Instagram to disclose their state of mind, which includes distress, need for assistance, vulnerability, happiness, etc. [9]. Out of the 500 Instagram posts analyzed using five different hashtags related to BD (#bipolar, #bipolardisorder, #bipolarawareness, #bipolarwarrior, and #bipolarrecovery), only 196 posts were found to be relevant, considering the inclusion and exclusion criteria. This suggests that a significant number of posts are irrelevant and unreliable. With 3,49,409 likes, 3,379 comments, and 5,125,083 total followers of the Instagram accounts that made the post, the 196 posts that were included in this study evidently had a vast audience.

In our study, out of 196 posts, only 4.6% of posts (9 posts) and 11.7% of posts (23 posts) were by medical professionals and healthcare organizations, respectively. A staggering amount of posts - 70.4% of posts (138 posts) - were posted by patients. This is in contrast to a study by Aiman et al., 2023, which shows 54.52% of posts (229 posts) on obesity were posted by the health and wellness industry/website, and only 13.81% of the posts (58 posts) were posted by the survivors or the patients [10]. This suggests that individuals with bipolar disorder or other mental illnesses are more engaging on social media platforms compared to other non-psychiatric illnesses like obesity, stroke, and diabetes. Findings from qualitative studies suggest that people with severe mental health conditions, such as BD, actively seek out ways to communicate with their peers online and that peer support naturally happens among individuals who share their experiences online. Research also points to the potential for social media to confer peer support to those with BD, either as a supplement or alternative to in-person support [11]. Having more healthcare professionals get involved in using social media platforms to share information about BD will help users find more relevant and reliable information about the disorder and reduce misconceptions on these platforms [12].

Amongst the top five hashtags included in our study related to BD, it is found that #bipolar and #bipolardisorder were used for only 12 and 16 posts, respectively, included in the study out of the 100 posts analyzed under these hashtags. This is the least number of posts included compared to other hashtags related to BD. Other hashtags analyzed, like #bipolarrecovery, #bipolarawareness, and #bipolarwarrior, were used in 45, 46, and 77 posts included, respectively, considering the total number of posts under #bipolar on Instagram as of August 23, 2023, 12:30 p.m., is 2,838,875. This suggests that the use of common hashtags like #bipolar or #bipolardisorder is more commonly done on Instagram but does not necessarily mean that the information provided on these posts is reliable and relevant to the BD. This spreads misinformation amongst the audiences of these posts. This may be seen as proof that hashtags give users a way to join groups outside of their direct contacts, especially for groups defined by mental conditions or rather uncommon interests [13, 14].

In our study, 121 posts (61.73%) had information about symptoms of BD, and 109 posts (55.61%) were about patients sharing their own experience regarding BD. In a study by Budenz et al., 2020, only 13% of the tweets in the sample contained personal experiences by the patients, but those tweets were much more likely to be retweeted. This shows that more retweets are done to make it more visible, thereby encouraging other users to disclose their mental health. This practice may improve psychological health and boost caretaking. Most BD tweets were expressions of emotional/esteem support, in which users wrote messages empathizing with others or encouraging self-efficacy [15,16]. In another study by Lee et al., 2020, 27.2% of Instagram posts (206 posts) that were analyzed featured a message from a person who testified about their experience with mental health [14].

In our study, among the posts that were analyzed, 81.6% (n = 160) posts were in the form of images and 62.24% (n = 122) were digitally created images/videos. Photos made up more than half of posts (54%, n = 409) while images with illustrative composition made up 46% (n = 249) of posts in a study by Lee et al., 2020 [14]. In our study, the median GQS of posts uploaded by medical professionals was 3, healthcare organizations was 2, and by patients was 2. In a similar study by Popoola-Samuel et al., 2023, that analyzed seizure disorder content on Instagram the difference in GQS was statistically significant (p = 0.0033) between Group A (healthcare professionals) and Group B (Non-healthcare professionals), the mean GQS of Group A (n = 200) was 2.42 and Group B (n = 231) was 2.13 [17].

Limitations

This is an exploratory and one of its kind study examining mental health disorder, specifically Bipolar Disorder content on Instagram. But there are certain limitations of this study which restrict the generalizability of its findings. One of the limitations is the sample size. Only top 500 posts, including 100 posts under each hashtags mentioned before were analyzed. Considering the vastly available user content data on Instagram, expanding the number of posts analyzed will help to provide a better and complete analysis on the quality and reliability of its content. Another limitation is the lack of involvement of posts generated by private accounts on Instagram, which are not accessible to the public. Lastly, the fact that people who do not use Instagram or the internet were not included in the research makes it difficult to generalize our results.

## Conclusions

Instagram, which is one of the most used applications, can be used as a source to share knowledge about BD and also communicate with the individuals experiencing symptoms and provide them with better management options. Using this platform will help healthcare professionals reach a wider number of audience. Further research should be done to find strategies to prevent spread of irrelevant and unreliable information of these social media platforms, thus enhancing user’s education and decision-making in the long term.
